# The Emergence of a Multidrug-Resistant and Pathogenic ST42 Lineage of Staphylococcus haemolyticus from a Hospital in China

**DOI:** 10.1128/spectrum.02342-21

**Published:** 2022-05-17

**Authors:** Mingyang Qin, Ping Chen, Baoguo Deng, Ruowen He, Yiping Wu, Yanxian Yang, Wenbin Deng, Xin Ding, Fan Yang, Chuanbo Xie, Yongqiang Yang, Guo-Bao Tian

**Affiliations:** a School of Basic Medical Sciences, Xinxiang Medical University, Xinxiang, China; b Department of Microbiology, Zhongshan School of Medicine, Sun Yat-sen University, Guangzhou, China; c Key Laboratory of Tropical Diseases Control (Sun Yat-sen University), Ministry of Education, Guangzhou, China; d Cancer Prevention Center, Sun Yat-sen University Cancer Center, Guangzhou, China; e State Key Laboratory of Oncology in South China, Guangzhou, China; f Collaborative Innovation Center for Cancer Medicine, Guangzhou, China; g School of Pharmaceutical Sciences (Shenzhen), Sun Yat-sen University, Guangzhou, China; h School of Medicine, Xizang Minzu University, Xianyang, Shaanxi, China; Hartford Hospital

**Keywords:** *Staphylococcus haemolyticus*, ST42, antimicrobial resistance, molecular epidemiology, virulence

## Abstract

Staphylococcus haemolyticus is an opportunistic pathogen associated with hospital-acquired infections. However, the genetic diversity of *S. haemolyticus* among the patients and the hospital environment is largely unknown. Here, we isolated 311 *S. haemolyticus* strains from different sampling sites of patients and hospital environment. Genomic analysis showed that ST42 is an emerging clone widely disseminated in the hospital. *S. haemolyticus* ST42 strains exhibited decreased susceptibilities for multiple antibiotics compared with other STs and carried significantly more antibiotic resistance genes (ARGs). Furthermore, ST42 strains harbored more virulence genes per isolate than in other STs, and the capsular biosynthesis genes *cap*DEFG were more prevalent in ST42 strains. Using the Galleria mellonella infection model, we demonstrated that ST42 strains are highly virulent compared with non-ST42 strains. Taken together, our data identified an emerging ST42 clone of *S. haemolyticus* with aggregated ARGs and virulence determinants in the hospital, representing a significant health threat in terms of both disease and treatment.

**IMPORTANCE** *S. haemolyticus* is an emerging opportunistic pathogen with a high burden of antimicrobial resistance. We performed molecular epidemiological analysis of *S. haemolyticus* that was isolated from a hospital, and found that the phylogenetic lineages are diverse accompanied by a dominant epidemic clonal lineage ST42. We demonstrated that *S. haemolyticus* ST42 strains have been disseminated among patients and the hospital environment. The data provide mechanistic insight and indicate that *S. haemolyticus* ST42 strains are multidrug-resistance and virulent clones via accumulating more ARGs and virulence genes.

## INTRODUCTION

Staphylococcus haemolyticus is considered the second most common species of coagulase-negative staphylococci (CoNS) and has been regarded as an important hospital-acquired pathogen (1). *S. haemolyticus* may cause septicemia, peritonitis, otitis, urinary tract infections, and respiratory infections (1, 2). However, the biological significances and genomic diversity of *S. haemolyticus* remain largely unknown. A previous study showed that clonal complex (CC) 29 was the main epidemiological lineage among the given data set (3). Limited evidence emphasized that *S. haemolyticus* is becoming more concerning due to its multidrug resistance (MDR, resistance to more than three classes of antibiotics.) (4–6). It is necessary to trace the emergence of MDR clones in hospitals. Furthermore, the extraordinary genome flexibility of *S. haemolyticus* has been identified to facilitate survival in the hospital environment and the acquisition of antibiotic resistance genes (ARGs), contributing to the dissemination of resistance genes to other nosocomial staphylococci (1, 7, 8). However, the phylogenetic relationship of *S. haemolyticus* between patients and the hospital environment has not been investigated.

Another concern of staphylococci infections is that some bacteria can produce biofilm for immune evasion to the hosts and defend the treatment, playing an important role in the pathogenicity of Staphylococcus spp. (9, 10). A previous study investigated *S. haemolyticus* strains that were isolated from bloodstream infections, and demonstrated that 34% of strains can form biofilm *in vitro* (11). The *S. haemolyticus* isolates from the infected eyes and healthy conjunctiva can also produce biofilm on polystyrene microtiter plates (12). Although *S. haemolyticus* is one of the most common CoNS species causing hospital-acquired infections, little is known about the complications between strain diversity and virulence determinants.

In this study, we prospectively collected samples from the patients and the hospital environment in a hospital cohort. We further isolated *S. haemolyticus* strains and conducted a large-scale genomic analysis of *S. haemolyticus* to clarify the phylogenetic relationship among multiple niches. We identified the ST42 lineage is the prevalent clone with a high burden of ARGs and virulence determinants that has been disseminated in multiple sources of the patients and the hospital environment, representing an emerging pathogenic clone of both disease and treatment.

## RESULTS

### Identification of *S. haemolyticus* from the patients and the ward environment.

In order to better clarify the distribution of *S. haemolyticus* in the hospital, samples were collected from multiple human resources and the hospital environment. A total of 311 *S. haemolyticus* isolates were recovered from the patient samples and the ward environment. Specifically, 176 isolates were from the ward environment, 83 from feces, 43 from nares, 7 from bronchoalveolar lavage (BAL) fluid, and 2 isolates from throats. Furthermore, the environmental sampling sites including the surface of the pillow, the surface of the quilt, the surface of safe guard, and the bedside. These data indicate that *S. haemolyticus* can colonize in the hospital environment, which can be a reservoir of *S. haemolyticus*.

### High genetic diversity of *S. haemolyticus* and an emerging ST42 clone disseminated in the hospital.

**FIG 1:**
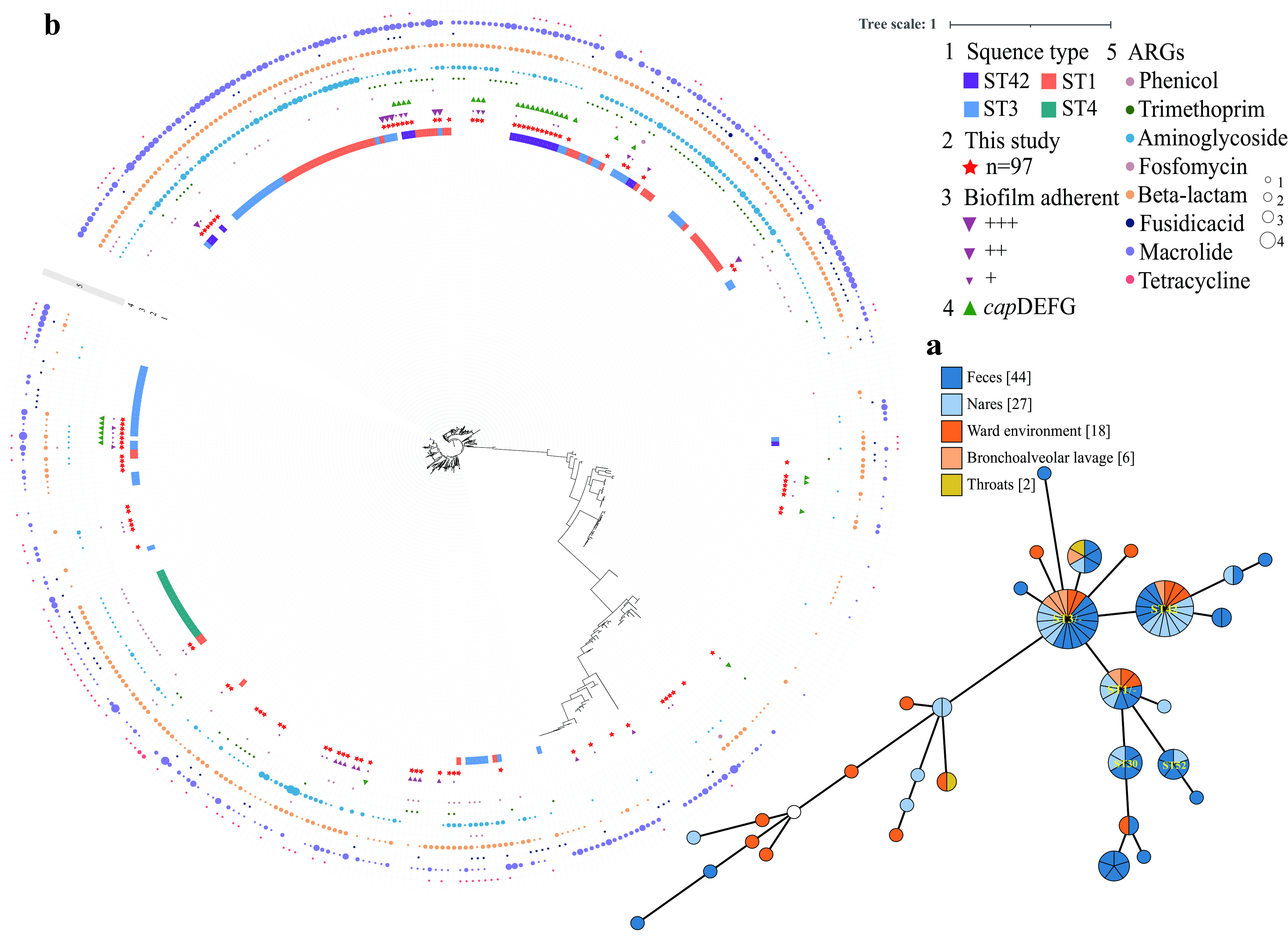
Population structure of *S. haemolyticus* isolates. (a) The minimum spanning tree was constructed on the basis of housekeeping genes, including *arcC*, *SH_1200*, *hemH*, *leuB*, *SH1431*, *cfxE*, and *Ribose_ABC*. Each node within the tree represents a ST or ST-like type, with diameters scaled to the number of isolates belonging to that type. The figure is colored by different sources of strains. (b) Phylogenetic analysis of *S. haemolyticus* strains from this study and publicly available sources. The first circle represents the different ST types, the second circle shows the strains in this paper, the third circle exhibits the biofilm-forming ability, the fourth circle represents the strains with the *cap* gene cluster, and others show the number of ARGs for different category antibiotics.

To clarify the molecular epidemiological characteristics of *S. haemolyticus*, we randomly selected 97 strains as a representative of multiple resources from the human and the hospital environment for whole-genome sequencing (WGS). A total of nine known STs were identified among 56.7% (55/97) sequenced isolates. The dominant clonal lineage is ST42 (*n* = 17), followed by ST3 (*n* = 14), ST1 (*n* = 8), ST30 (*n* = 6), and ST52 (*n* = 5). Furthermore, the remaining 42 strains belong to unidentified ST types with new alleles, which include 22 from feces, 6 from nares, 12 from the ward environment, 1 from BAL, and 1 from throat swab (Table S1 in the supplemental material), representing a high genetic diversity of our data set. By uploading new alleles, we identified 30 new multilocus sequence types (MLSTs), ST 96–122, and the remaining four strains have not been identified (Table S2). ST42 strains were identified from multiple resources, including nares, feces, BAL fluid, and the ward environment. By constructing a minimum spanning tree (MST) of the isolates, the dominant STs were separated from each other and recovered from diverse sources (Fig. 1a). We performed cluster analysis of clonal complexes based on the housekeeping genes of MLST that have been identified in 433 strains of public databases, and ST42 strains were classified into clonal complex 29 (Fig. S1). To investigate the phylogenetic relationship of *S. haemolyticus* at a higher resolution we constructed a phylogenetic tree by maximum likelihood approach based on the core genome single nucleotide polymorphisms (cgSNPs). Additionally, all the publicly available *S. haemolyticus* were enrolled as a comparison as of 1 June 2021, leading to a large-scale collection of 433 *S. haemolyticus* genomes for analysis (Table S3). The results revealed a deep branching and scattered population structure that was broadly classified into distinct phylogenetic lineages, representing a high genetic diversity of the *S. haemolyticus* species (Fig. 1b). Furthermore, the population structure of the newly sequenced isolates was highly mixed with the reference genomes. Among the prevalent STs, strains belonging to ST4 were all clustered together while a scattered phylogeny was observed for ST1, ST3, and ST42, indicating high genomic plasticity within those clones. Besides, the ST42 strains were continuously isolated from January to November 2019.

### *S. haemolyticus* ST42 strains exhibited extensive MDR profiles.

Antibiotic susceptibility testing of the 97 *S. haemolyticus* isolates showed that 75.3% (73/97) were MDR strains. There were 16 strains that were resistant to three classes of antibiotics, 21 were resistant to four classes of antibiotics, 17 were resistant to five classes of antibiotics, 16 were resistant to six classes, and 3 were resistant to seven classes of antibiotics. All of the isolates displayed a common antimicrobial susceptibility profile, showing resistance to various classes of antibiotics. The highest resistance rate was observed for ciprofloxacin (86.6%, 84/97), followed by oxacillin and cefotaxime (83.5%, 81/97), erythromycin (79.4%, 77/97), gentamicin (56.7%, 55/97), tetracycline (40.2%, 39/97), the resistance rate of clindamycin and rifampicin were <30%. On the contrary, all strains were sensitive to vancomycin and linezolid. This indicated that *S. haemolyticus* is a species with extensive MDR profiles.

**FIG 2:**
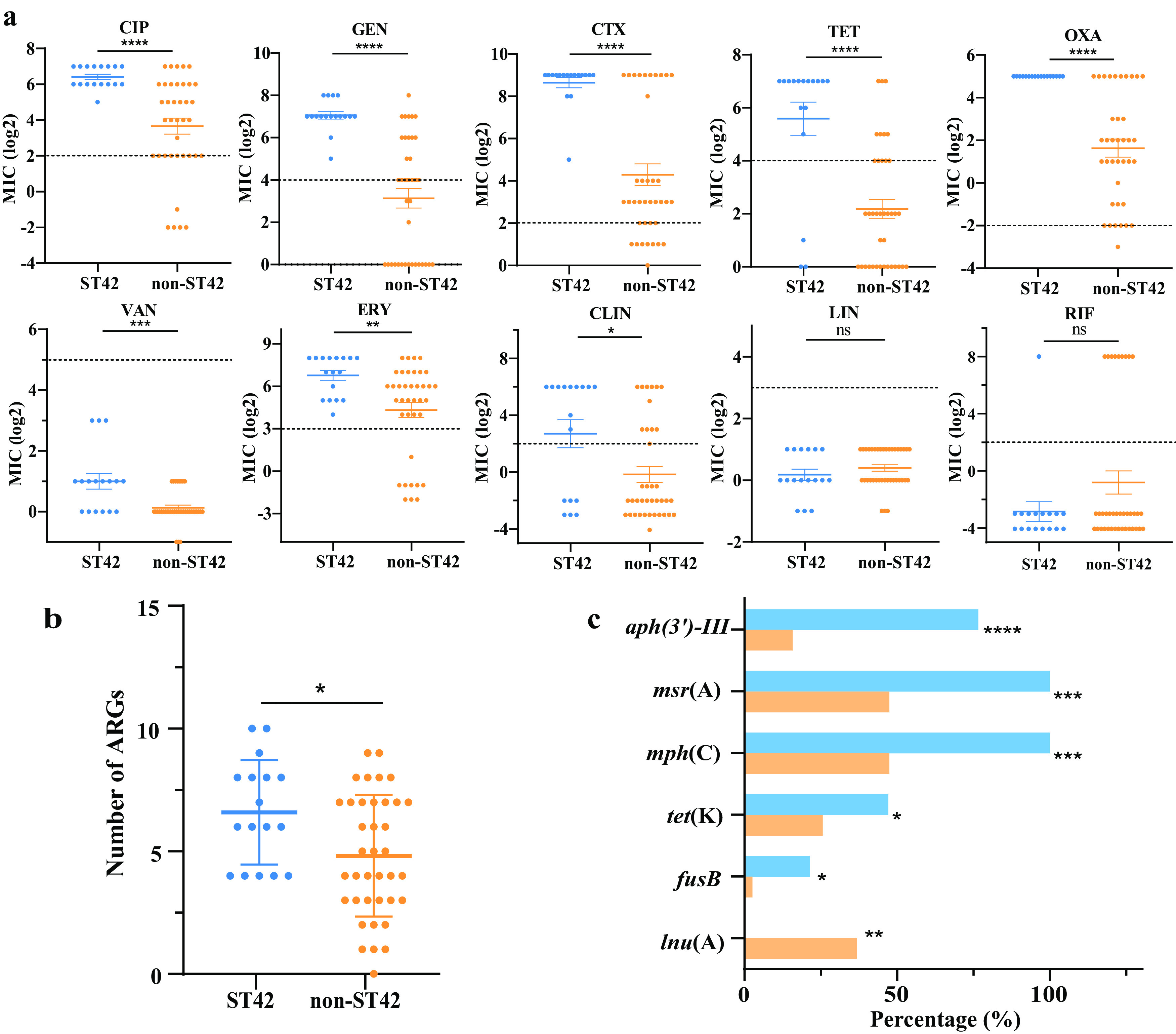
Antimicrobial susceptibility testing and antibiotic resistance genes (ARGs) between ST42 and non-ST42 strains. (a) The MICs were log-transformed for statistical analysis. Dashed lines represent resistance breakpoints (*t* test, *, *P* < 0.05; ****, *P* < 0.0001; ns,*P* > 0.05; the error bars were SEM). (b) The average number of ARGs per strain. The average gene number of ST42 strains is significantly higher than that of non-ST42 strains (*t* test, *, *P* < 0.05 error bar was SEM). (c) Prevalence of different ARGs between ST42 and non-ST42. The carrying rate of *aph(3′)-III*, *msr*(A), *mph*(C), *tet*(K), and *fus*(B) genes in ST42 strains (blue) is higher than that in non-ST42 strains (orange) (Chi-square test, *, *P* < 0.05; **, *P* < 0.01; ***, *P* < 0.001; ****, *P* < 0.0001).

Furthermore, it showed that ST42 strains exhibited greater MDR profiles, and all the strains developed resistance to ciprofloxacin, gentamicin, cefotaxime, erythromycin, and oxacillin (Table 1). Since ST42 was the dominant known ST (17/57), we made a comparison between ST42 and other ST types (STs). The susceptibility testing among all the ST42 isolates revealed significantly high MICs for multiple antimicrobial agents, namely, ciprofloxacin, gentamicin, cefotaxime, erythromycin, oxacillin, tetracycline, clindamycin, and vancomycin (*P < *0.05) (Fig. 2a). These results indicated that ST42 was an emerging lineage with a high burden of antimicrobial resistance. Among the different sources of *S. haemolyticus* isolation, we observed differences in MICs for several antibiotics between strains of nares and the ward environment. Specifically, the MICs values of nasal strains to ciprofloxacin and clindamycin were significantly higher than those of environmental strains (Fig. S2a).

**TABLE 1:** The antimicrobial susceptibility testing result of ST42 *S. haemolyticus* strains^*a*^

ID	Source	MLST	ARGs	MIC (μg/mL)									
				CIP (100%)	GEN (100%)	CTX (100%)	ERY (100%)	RIF (0.06%)	TET (82.4%)	OXA (100%)	CLIN (58.8%)	VAN (0%)	LIN (0%)
SYSUSHA_122	Nares	ST42	*aac(6′)-aph(2′')*, *aph(3′)-III*, *blaZ*, *dfrG*, *erm*(C), *mecA*, *mph*(C), *msr*(A)	**128**	**128**	**>256**	**>128**	0.125	**128**	**>16**	**>32**	2	2
SYSUSHA_121	Nares	ST42	*aac(6′)-aph(2′')*, *aph(3′)-III*, *blaZ*, *dfrG*, *erm*(C), *mecA*, *mph*(C), *msr*(A)	**64**	**32**	**32**	**128**	0.125	**128**	**>16**	**16**	1	2
SYSUSHA_79	Nares	ST42	*aph(3′)-III*, *blaZ*, *mph*(C), *msr*(A)	**128**	**128**	**>256**	**128**	0.06	**128**	**>16**	**>32**	1	1
SYSUSHA_78	Feces	ST42	*aph(3′)-III*, *blaZ*, *mph*(C), *msr*(A)	**128**	**128**	**>256**	**128**	0.06	**128**	**>16**	**>32**	1	1
SYSUSHA_76	Nares	ST42	*aph(3′)-III*, *blaZ*, *mph*(C), *msr*(A)	**64**	**128**	**>256**	**>128**	0.06	**128**	**>16**	**>32**	2	1
SYSUSHA_75	Nares	ST42	*aph(3′)-III*, *blaZ*, *mph*(C), *msr*(A)	**128**	**128**	**>256**	**>128**	0.06	**128**	**>16**	**>32**	1	1
SYSUSHA_49	Ward environment	ST42	*aph(3′)-III*, *blaZ*, *mecA*, *mph*(C), *msr*(A), *tet*(K)	**64**	**128**	**>256**	**16**	0.06	**128**	**>16**	0.125	2	1
SYSUSHA_31	Nares	ST42	*aph(3′)-III*, *blaZ*, *mph*(C), *msr*(A)	**64**	**128**	**256**	**32**	0.06	**32**	**>16**	0.125	2	0.5
SYSUSHA_16	Feces	ST42	*aph(3′)-III*, *blaZ*, *fusB*, *mph*(C), *msr*(A)	**128**	**256**	**>256**	**>128**	0.125	**128**	**>16**	**>32**	2	2
SYSUSHA_13	Feces	ST42	*aac(6′)-aph(2′')*, *aph(3′)-III*, *blaZ*, *erm*(C), *mecA*, *mph*(C), *msr*(A), *sμL*, *tet*(K)	**128**	**128**	**>256**	**>128**	0.125	**128**	**>16**	0.25	2	2
SYSUSHA_123	Nares	ST42	*aac(6′)-aph(2′')*, *aph(3′)-III*, *blaZ*, *mecA*, *mph*(C), *tet*(K)	**128**	**128**	**>256**	**32**	0.125	**128**	**>16**	0.25	2	2
SYSUSHA_111	Nares	ST42	*aac(6′)-aph(2′')*, *aph(3′)-III*, *blaZ*, *dfrG*, *erm*(C), *fusB*, *mecA*, *mph*(C), *msr*(A), *tet*(K)	**64**	**>256**	**>256**	**>128**	0.125	**64**	**>16**	**>32**	8	0.5
SYSUMLU_2	Ward environment	ST42	*aac(6′)-aph(2′')*, *aph(3′)-III*, *blaZ*, *dfrG*, *erm*(C), *fusB*, *mecA*, *mph*(C), *msr*(A), *tet*(K)	**64**	**>256**	**>256**	**>128**	0.125	**64**	**>16**	**>32**	8	0.5
SYSUGLS028	Ward environment	ST42	*aph(3′)-III*, *blaZ*, *mecA*, *mph*(C), *msr*(A), *tet*(K)	**128**	**128**	**>256**	**32**	0.125	**128**	**>16**	0.25	2	2
SYSUSHA_81	Feces	ST42	aac(6′)-aph(2′'), aph(3′)-III, blaZ, dfrG, erm(C), mecA, mph(C), msr(A)	**32**	**256**	**>256**	**>128**	**>128**	≤1	**>16**	>32	8	1
SYSUSHA_6	BAL	ST42	*aac(6′)-aph(2′')*, *aph(3′)-III*, *blaZ*, *mecA*, *mph*(C), *msr*(A)	**64**	**128**	**>256**	**32**	0.06	2	**>16**	0.125	1	1
SYSUSHA_36	Feces	ST42	*aac(6′)-aph(2′')*, *aph(3′)-III*, *blaZ*, *erm*(C), *mecA*, *mph*(C), *msr*(A), *vga*(A)LC	**64**	**64**	**>256**	**64**	0.06	≤1	**>16**	**8**	1	1

a MLST, multi-locus sequence typing; MIC, MICs; BAL, bronchoalveolar lavage; ARGs, antibiotic resistant genes; CIP, ciprofloxacin (Bp 4 μg/mL); OXA, oxacillin (Bp 0.5 μg/mL); CTX, cefotaxime (Bp 4 μg/mL); ERY, erythromycin (Bp 8 μg/mL); RIF, rifampicin (Bp 4 μg/mL); GEN, gentamicin (Bp 16 μg/mL); TET, tetracycline (Bp 16 μg/mL); CLIN, clindamycin (Bp 4 μg/mL); VAN, vancomycin (Bp 32 μg/mL); LIN, linezoid (Bp 8 μg/mL); Bp, Breakpoint. The percentage means the resistant isolates rate to each antibiotic. Bold type means resistant value.

### *S. haemolyticus* ST42 strains harbored more ARGs than in other STs.

Through WGS data, a total of 23 ARGs were identified among the 97 *S. haemolyticus* isolates (Fig. S2b), conferring resistance to nine different antibiotics (Fig. 1b). Some prevalent genes include *mecA* (56/97, 57.7%, encoded penicillin-binding protein 2), *blaZ* (77/97, 79.4%, encoded classA β-lactamase), *msr*(A) (56/97, 57.7%, encoded ABC-F type ribosomal protection protein), *mph*(C) (50/97, 51.5%, encodes Mph(C) family macrolide 2′-phosphotransferase), *aac(6′)-aph(2′')* (40/97, 41.2%, encoded aminoglycoside N-acetyltransferase and O-phosphotransferase), *aph(3′)-III* (32/97, 33%, encoded aminoglycoside O-phosphotransferase APH(3′)-III), *erm*(C) (28/97, 28.9%, encoded 23S rRNA [adenine(2058)-N(6))-methyltransferase Erm(C)]), *tet*(K) (23/97, 23.7%, encoded tetracycline efflux MFS transporter Tet(K)).

We further explored the differences in the prevalence of ARGs between ST42 and non-ST42 isolates. The average number of ARGs per isolate carried by ST42 strains was significantly higher than in other ST strains (Fig. 2b). It showed that there were significant differences among *aph(3′)-III*, *msr*(A), *mph*(C), *tet*(K), and *fus*(B) (encoded fusidic acid resistance EF-G-binding protein) genes, and the prevalence of the genes in ST42 strains were significantly higher than that of other ST strains (Fig. 2c). However, the carrying rate of the *lnu*(A) (encoded lincosamide nucleotidyltransferase) gene in non-ST42 strains was significantly higher than in ST42 strains.

### The high burden of virulence determinants was prevalent in *S. haemolyticus* ST42 strains.

**FIG 3:**
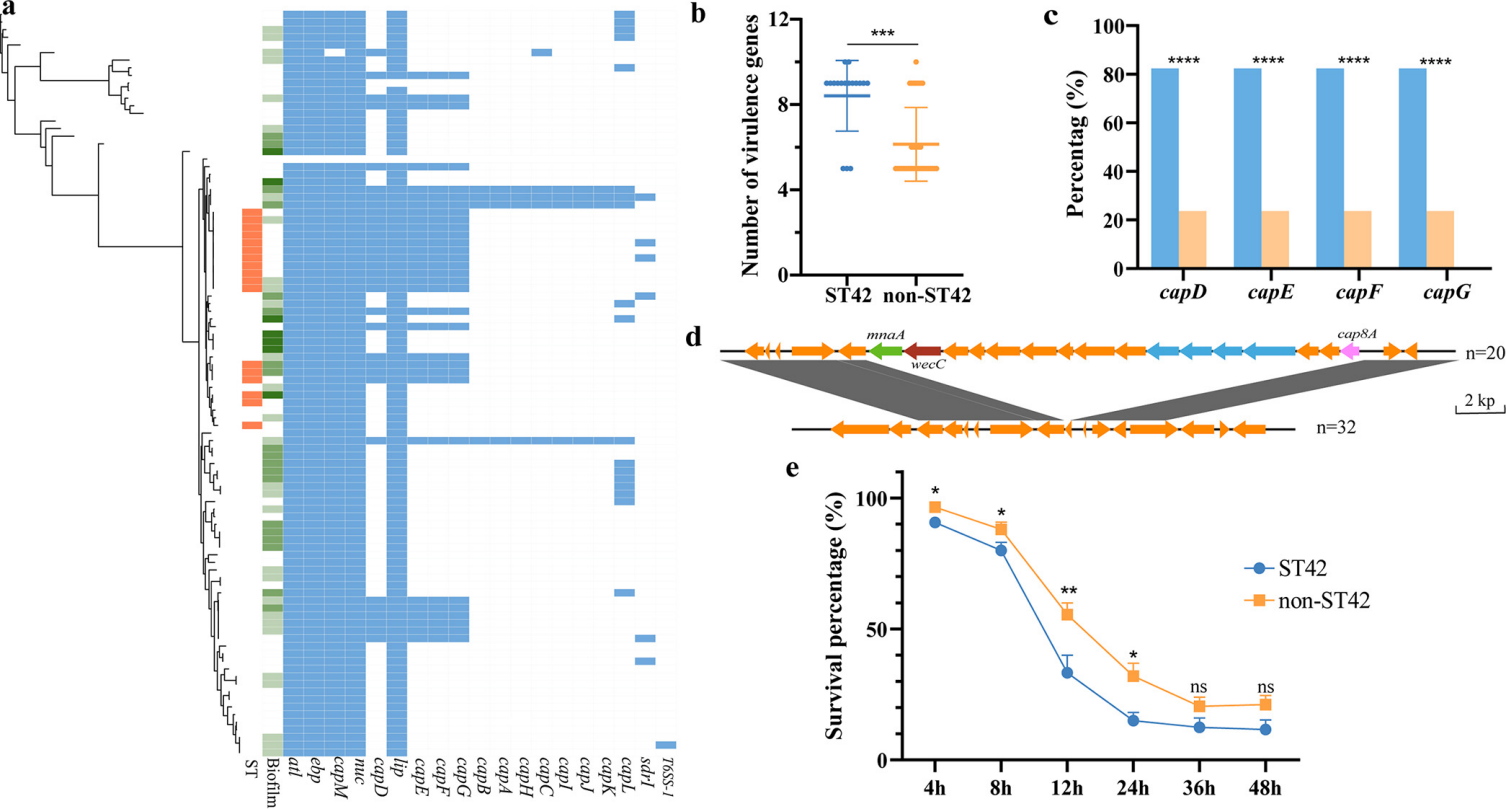
Virulence determinants of *S. haemolyticus* isolates. (a) A maximum likelihood phylogeny tree was constructed by the 97 sequenced isolates and a reference strain. The distribution of 19 identified virulence genes is shown by a heat map. Orange color shows strains with ST42 type. Green color exhibits biofilm formation ability of the strains, the light color is weakly biofilm adherent, and the strong color is strong biofilm adhesion. Colors between the two preceding colors indicate moderate biofilm adhesion. (b) The total number of virulence genes was carried by each strain. The statistical analysis of comparison was conducted by *t* test (*, *P* < 0.05). (c) Comparative analysis of virulence factors. ST42 (blue) and non-ST42 (orange) by Chi-square, ****, *P* < 0.0001. (d) The genetic environment of the *cap*DEFG gene cluster (blue), *cap8A* (pink), *wecC* (red), and *manA* (green). Arrows show the direction of transcription of open reading frames. Dark gray shadows indicate 100% homology similarity. (e) The statistical analysis of the survival of G. mellonella infected with ST42 and non-ST42 strains, which was monitored for 48 h postinjection with observations. Each point presents the average of the results obtained with survival rate of the 17 strains of the ST42 (Mann-Whitney test, *, *P* < 0.05; **, *P* < 0.01). The points were average and the error bars were SEM.

A total of 19 virulence genes were identified among the 97 isolates. *atl* (autolysin), *ebp* (cell surface elastin binding protein EbpS), *nuc* (nuclease), *lip* (lipase correlation), and *capM* (capsule synthesis correlation) were prevalent among almost of all strains, except for one *lip* negative isolate and one *capM* negative isolate (Fig. 3a). A previous study has reported that *nuc* DNA load was significantly elevated in patients with sepsis (13). Another recent report has found that the Lipase1 enhanced virus replication (14), suggesting an association of *lip* with virulence. Moreover, the average number of virulence genes carried by ST42 strains was significantly higher than that carried by other STs (Fig. 3b). In addition, comparing the carrying rate of all virulence factors showed that *capD*, *capE*, *capF*, and *capG* genes were more prevalent in ST42 (14/17, 82.4%) than in STs strains (9/38, 23.7%) (Fig. 3c). The *cap* genes are involved in capsular polysaccharide biosynthesis and contribute to antibiotic resistance and infection (15, 16). We further analyzed the genetic environment of the *cap*DEFG genes, and we found that they were presented as a gene cluster and located together. Furthermore, we observed that the sequences without the *cap* genes also lacked a genomic fragment containing the *cap* cluster. The fragment was 17,722 bp and contained 17 open reading frames. These included genes *cap8A* upstream of *cap* gene cluster, which encoded capsular polysaccharide type 8 biosynthesis protein Cap8A, and *wecC* with *manA* downstream of the *cap* gene cluster, both of which encoded products associated with the biosynthesis of teichoic acid in bacterial cell walls (Fig. 3d) (17).

### *S. haemolyticus* ST42 strains tend to be a highly pathogenic clone.

Previous study has reported that *S. haemolyticus* was the most virulent species among CoNS (18). To confirm this, we investigated the pathogenicity of *S. haemolyticus* strains using a G. mellonella infection model. There were 17 ST42 strains, 8 isolates from nares source, 5 from feces, 3 environmental strains, and 1 strain from BAL. And there were 32 non-ST42 strains, 14 isolates from feces, 12 from nares, 3 from BAL, 2 environmental strains, and 1 strain from throats. It showed that the survival rate of animals infected by ST42 strains was significantly lower than in other ST strains within 24 h, with a peak of 12 h (Fig. 3e). Furthermore, the percentage of surviving larvae was lower following infection with *S. haemolyticus* strains compared to the virulent S. aureus ATCC29213 at 12 and 24 h (Fig. S3b). Besides, it showed that the larval mortality of ST42 strains has reached about 65% at 12 h, and 85% lethality at 24 h, which were higher than other ST groups (Fig. 3e). These results indicated that *S. haemolyticus* was a virulent species and ST42 might be a highly pathogenic clone.

### Biofilm formation characteristics of *S. haemolyticus* strains.

We tested three different media to measure biofilm formation, including tryptic soy broth (TSB), TSB with 1% glucose (TSB_glu_), and 3% NaCl (TSB_NaCl_). We found that the biofilm formation ability of the strains was enhanced in the TSB_glu_, whereas the biofilm formation ability of the strains was weakened in the TSB_NaCl_. Furthermore, we observed that the 18 strains that did not produce biofilm in TSB alone changed to produce biofilm after glucose was added (Table S4). Furthermore, 35.3% (6/17) ST42 strains can form a biofilm under TSB medium, 58.8% ST42 strains can form a biofilm under TSB_glu_ medium, and the same as 29.4% strains can form a biofilm TSB_NaCl_ culture medium. Besides, there was no significant difference in biofilm-forming ability between ST42 and non-ST42 strains under TSB (Fig. S3a).

## DISCUSSION

Previous studies have reported that *S. haemolyticus* caused pneumonia (2), and in this study, we found that *S. haemolyticus* was enriched in the patients with lower respiratory infection. This prompted us to investigate the molecular characteristics of *S. haemolyticus* across the hospital niches at the strain level. Indeed, we found that the prevalent ST42 clones were disseminated across different sites of the patients and the ward environment. We next performed a large-scale genomic analysis by sequencing 97 strains and enrolled all the publicly available genomes. Excluding our strains, there were 336 strains from the public data: 233 were isolated from humans, 44 from animals, and 33 from the environment and others, and the remaining strains lacked sources (Table S3). Our result demonstrated the *S. haemolyticus* has a high genetic diversity, which is consistent with previous studies, suggesting that the catheter- or wound- or environment-derived *S. haemolyticus* genome has a high plasticity (19, 20). Furthermore, we found that ST42 was classified as CC 29, just like ST1, ST2, ST3, ST8, ST9, ST22, ST26, ST29, ST30, ST31, ST32, and ST33 indicated in a previous study (3). Besides, in the analysis of all available public data, we found that the most dominant clone was ST3, followed by ST1, and these strains were also found in our strain collection. A recent study indicated the outbreak of ST42 clones that collected from burn and other patients from a hospital in Taiwan and suggested ST42 may be related to ST3 (21). Our data provide novel information that *S. haemolyticus* ST42 is an emerging clone that has been disseminated in the hospital.

Antibiotic resistance is a global public health concern, and the antibiotic-resistant bacteria are emerging from different ecological niches (20). We found that 75.3% (73/97) of *S. haemolyticus* were MDR strains. This result is consistent with previous studies, which reported that about 70% of clinical and commensal *S. haemolyticus* strains were MDR (19, 22). Furthermore, ST42 strains were resistant to multiple antibiotics, particularly high resistance phenotypes to oxacillin and clindamycin, which are especially used to treat staphylococcal infections. And our study showed that *S. haemolyticus* had higher drug resistance rates to ciprofloxacin, oxacillin, and erythromycin, which was similar to the results of previous studies (4, 19, 20). The high MIC value of the ST42 strains is indeed directly related to some ARGs, for example, *aph(3′)*-*III* directly gives the strains resistance to aminoglycoside antibiotics, *msr*(A) and *mph*(C) are related to resistance to macrolides, and *tet*(K) and tetracycline are related. However, there are still resistance phenotypes independent of ARGs. Besides, we found that ST42 strains harbored more genes than in other ST strains. These data confirmed that *S. haemolyticus* is a MDR species and the emerging ST42 strains present a higher burden of antimicrobial resistance.

The previous studies have reported that clinical *S. haemolyticus* is the most virulent CoNS species (18). In this study, we demonstrated that all the tested *S. haemolyticus* strains were virulent. For ST42 strains, they harbored more virulence genes than other ST strains. The examples include the high prevalence of *cap*DEFG in ST42 strains, and 125 of 336 public strains have the *cap* gene cluster. Using the G. mellonella infection model, we found that the survival rate of the ST42 group was significantly lower than that of STs in the first 24 h. Besides this, the survival percentage of ST42 at 12 and 24 h was also lower than that of virulent S. aureus ATCC29213. Furthermore, biofilm formation was previously found to enhance the colonization of human pathogens (23), and 56.7% of strains in this study can form biofilm under TSB medium. In the TSB_glu_, there were six strains that did not produce biofilm under TSB were capable of producing biofilms. This result suggests that the biofilm-forming ability of ST42 strains can be promoted in the presence of a certain amount of glucose. We concluded that *S. haemolyticus* ST42 is a highly pathogenic clone with a high burden of virulent determinants.

According to our data, biofilm formation by *S. haemolyticus* was enhanced when the organism is cultivated in TSB_glu_ (Table S4). This result is consistent with previous studies, which demonstrated glucose can promote the biofilm formation of *S. haemolyticus* (12). The information about *S. haemolyticus* virulence factors is scarce. Studies have shown that the biofilm formation of *S. haemolyticus* is independent of the *ica* gene, which was also confirmed by our strains not carrying *ica* (11). Furthermore, the mechanism of biofilm formation has not been elucidated. A few studies showed that the biofilm adhesion of *S. haemolyticus* was related to extracellular DNA, and the *strA* gene was found to play a role in the initial development of biofilm (12, 24). However, the *strA* gene was not detected in our strains, and the regulation of biofilm formation should be further studied. Besides, among the non-ST42 strains, there were two strains with strong biofilm formation ability, carrying 18 and 17 virulence genes respectively, which may be one of the reasons contributing to the high yield of biofilm.

In conclusion, we clarified the genomic diversity of *S. haemolyticus* from the patients and the ward environment and found that ST42 is an emerging clone that is disseminated in the hospital. We demonstrated that ST42 strains were highly pathogenic and MDR phenotypes, supported by the carriage of enriched virulent determinants and ARGs. The prevalence of the *S. haemolyticus* ST42 clone represents a significant health threat in terms of both disease and treatment. The epidemiology and molecular characteristics of *S. haemolyticus*, especially for ST42 strains in the hospitals, need to be further investigated.

## MATERIALS AND METHODS

### Sample collection and bacterial strains.

This study was conducted in a 1,600-bed tertiary hospital in Guangdong province, China, and approved by the Research Ethics Committee of the hospital (approval number K51-2, 2018). Hospitalized patients diagnosed with acute low respiratory tract infection from 15 December 2018 to 15 January 2020 were assessed for eligibility. The nasal swab, throat swab, and rectal swab were taken from participants using sterile cotton swabs. The bronchoalveolar lavage (BAL) fluid was collected via bronchoscopy. The ward environmental sampling was performed using sterile cotton swabs and cultured on sheep blood agar for 24 h under aerobic condition. Then the clones of different morphologies were selected and separated on mannitol salt agar two times, and finally confirmed as *S. haemolyticus* species by MALDI-TOF-MS (Bruker Daltonics, Germany). Specifically, we picked individual colonies with a sterile tip and smeared a thin film onto a MALDI target plate. The microbial films were then overlaid with a MALDI matrix selected as recommended by the MALDI-TOF manufacturers, typically 2.5 mg Bruker HCCA dissolved in 250 μL acetonitrile–water–trifluoroacetic acid (50:47.5:2.5). In our laboratory, to increase identification yield, we routinely add 70% formic acid on smeared microorganisms before adding (25).

### Antimicrobial susceptibility testing.

Antimicrobial susceptibilities of the strains were measured by determining the MICs of oxacillin (Penicillins), vancomycin (Glycopeptides), cefotaxime (Cephems), erythromycin (Macrolides), clindamycin (Lincosamides), gentamicin (Aminoglycosides), rifampicin (Ansamycins), tetracycline (Tetracyclines), ciprofloxacin (Fluoroquinolones), and linezolid (Oxazolidinones) using the agar dilution method, following the Clinical and Laboratory Standards Institute (CLSI) guidelines (26). Briefly, the monoclonal isolates were selected and cultured in tryptic soy broth (TSB) medium overnight (14–16h, 37°C, 200 rpm). Then, we used normal saline to adjust the bacteria to 0.5 MCF and used a multipoint inoculation instrument to inoculate the cells on Mueller-Hinton Agar (Oxiod, CM0337) containing antibiotics, and incubated for more than 16 h. The Staphylococcus aureus ATCC29213 strain was a control, and the experiments were repeated three times.

### Whole-genome sequencing and genotyping.

A total of 97 *S. haemolyticus* isolates from the contributing hospital were selected for WGS. Specifically, 44 strains were from feces, 27 from nares, 18 isolates were from the ward environment, 6 from BAL fluid, and 2 isolates from throats. DNA was extracted by the cetyltrimethylammonium bromide method. DNA libraries were constructed with 350-bp paired-end fragments (NEB kit) and sequenced using an Illumina HiSeq 2000 platform (Illumina kit). Short-read sequence data were *de novo* assembled using SPAdes v3.10 (using default parameters: –careful) (27). FastQC was used for data quality control (http://www.bioinformatics.babraham.ac.uk/projects/fastqc/). The coverage of the genomes was >100×. The MLST were identified using the BIGSdb (//bigsdb.web.pasteur.fr/staphylococcus/). The minimum spanning tree (MST) was constructed by GrapeTree (28). The eBURST approach was used for cluster analysis of MLST data and clonal complexes were defined as previously described (29) using geoBURST (http://www.phyloviz.net/goeburst/). ARGs and virulence genes were identified using ABRicate version 0.5 (https://github.com/tseemann/abricate) by aligning genome sequences to the ResFinder database (30) and VFDB database (31). New STs were identified by using PubMLST (https://pubmlst.org/) (32).

### Phylogenetic analysis.

For each *de novo* assembly, coding sequences were predicted using Prodigal v2.6 (33) and annotated using Prokka v1.13.3 (34). Core genes were identified and used to build the core genome using Roary v3.12 (35) with the -e- mafft setting to create a concatenated alignment of core genomic CDS. SNP-sites (https://github.com/sanger-pathogens/snp-sites) was used to extract the core-genome SNPs (cgSNPs) (36). To construct a maximum likelihood (ML) phylogeny of the sequenced isolates, RAxML v8.2.10 was used with the generalized time-reversible model and a GTRGAMMA distribution to model site-specific rate variation (37). We used iTOL to visualize and edit the phylogenetic tree (38). The phylogenetic tree is rootless. We evaluated the confidence degree according to the bootstrap method for 100 times, the iterations of the branches obtained all reached more than 50 times, and the branch was considered credible when its self-development support rate reached 50%.

### Biofilm formation.

Biofilm production *in vitro* was detected using the previously reported methods with minor modifications (12, 23, 39). Briefly, we adjusted the overnight cultures to OD600 = 0.2 (about 5 × 10^6^ CFU/mL) and added the diluted 20 μL bacterial cells to a 96-well plate that contained 180 μL fresh culture medium per well including TSB, TSB with 1% glucose (TSB_glu_), or 3% NaCl (TSB_NaCl_). Then, the cells were incubated at 37°C for 24h. After discarding the cell suspension, the plate was washed with phosphate-buffered saline (PBS) three times and dried thoroughly. The plate was fixed with 200 μL of methanol and stained with 1% crystal violet. Next, the bound dye was dissolved with 200 μL of 30% glacial acetic acid, and the solution OD was measured at 570 nm using a microplate reader (BioTek, Epoch2). The cutoff optical density (ODc) was defined as the mean OD of the negative control (medium only) (23). Based on the ODs of the bacterial films, all strains were classified into the following categories: nonadherent (0: OD ≤ ODc), weakly adherent (+: ODc < OD ≤ 2× ODc), moderately adherent (++: 2× ODc < OD ≤ 4× ODc), or strongly adherent (+++: 4× ODc < OD). The tests were triple.

### Galleria mellonella infection model.

The virulence of target strains was determined using the wax moth (G. mellonella) larvae model (40). Briefly, monoclonal isolates were selected and cultured in TSB liquid medium overnight (37°C, 200 rpm). Three doses of 1 × 10^5^, 1 × 10^6^, and 1 × 10^7^ CFU each with 10 worms per group were tested, and the dose of 1 × 10^6^ CFU was selected for the final test. We injected the cells into the left lower abdomen of the larva, with the needle at 45°and inserted 2–3 mm. The controls included the group that was injected with sterile PBS, S. epidermidis ATCC12228, and S. aureus ATCC29213 (1 × 10^6^ CFU), respectively. The larvae were incubated at 37°C in a darkroom and the survival rate was recorded for 48 h.

### Data availability.

The sequencing data generated and analyzed during the current study are available in the NCBI database BioProject no PRJNA783189. Links to genomic data of unknown ST types submitted to PubMLST are found in Table S5 in the supplemental material.
