# Supplementary material for: The Emergence of a Multidrug-Resistant and Pathogenic ST42 Lineage of Staphylococcus haemolyticus from a Hospital in China
Source: Microbiol Spectr. 2022 May 17;10(3):e02342-21. doi: 10.1128/spectrum.02342-21 (PMC9241665; doi:10.1128/spectrum.02342-21)
Supplement: SUPPLEMENTAL FILE 1 — Fig. S1 to S3; Tables S1, S2, and S4. Download spectrum.02342-21-s001.pdf, PDF file, 0.8 MB [file spectrum.02342-21-s001.pdf]

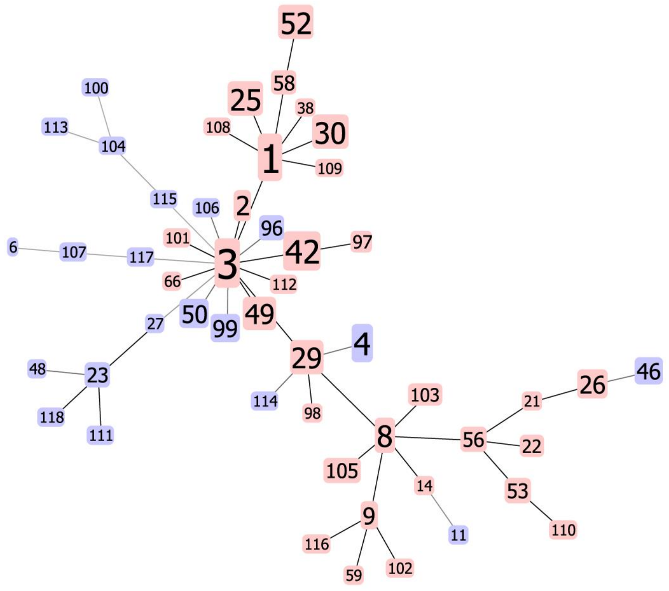

FIG S1 Clustering of clonal complexes. The numbers in the box represent ST type, and the pink showed clonal complex (CC) 29, and the remaining are other CCs.

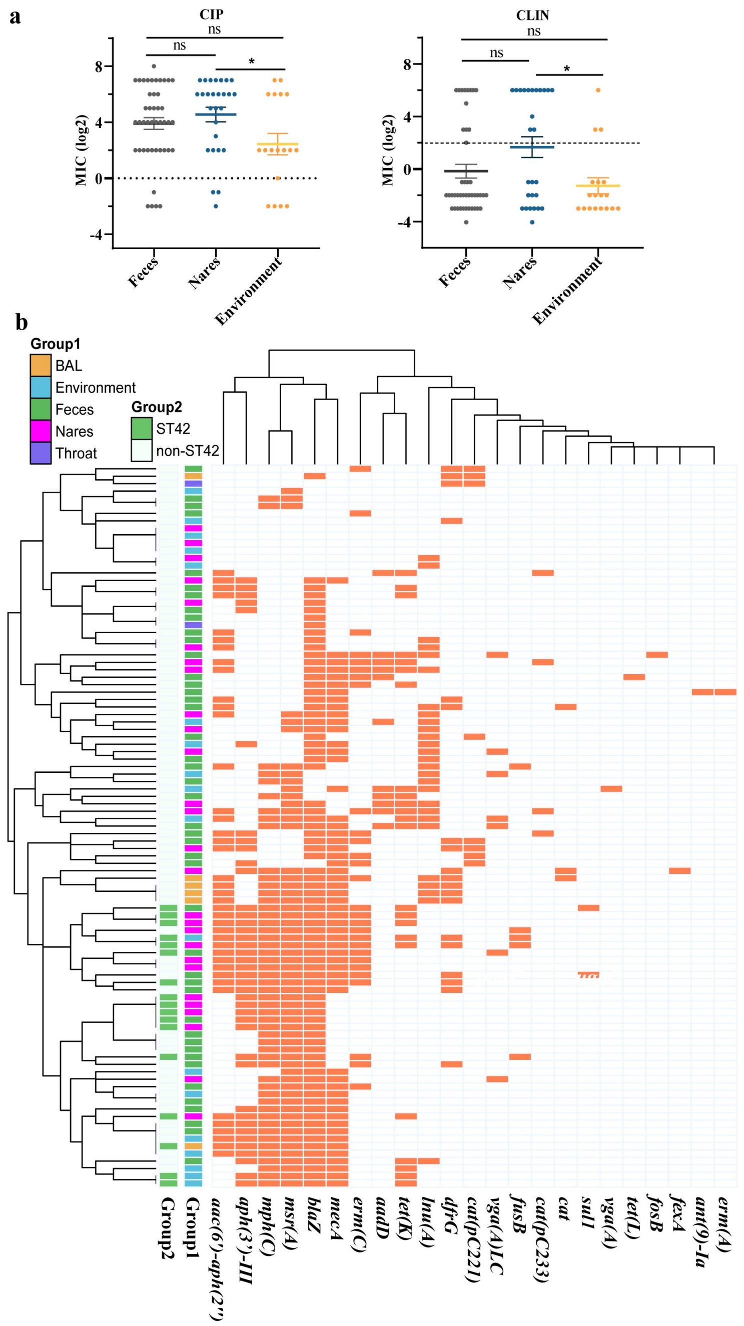

FIG S2 The comparison of MIC values of strains from different sources and the distribution of ARGs. (a) The MICs of CIP and CLIN between nares and ward environment were significantly difference (t-test, *P < 0.05, ns: p > 0.05). **(b)** Distribution of 23 ARGs among 97 *S. haemolyticu* strains. The color of group 2 was green is ST42 strains. Group 1 showed different sources of 97 strains. Yellow exhibited the presence of ARGs.

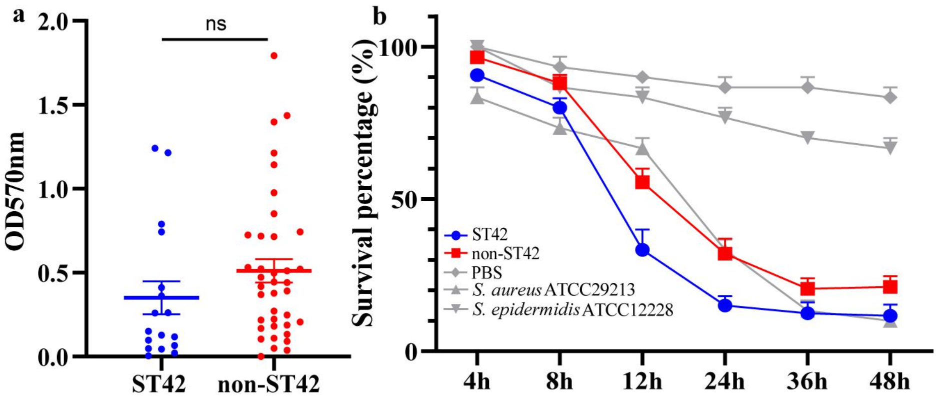

FIG S3 The biofilm formation and the pathogenicity of strains. (a) The comparison of OD570nm values between ST42 and non-ST42 strains. **(b)** Survival curves of *G. mellonella* for 48h mortality following *S. haemolyticus* infections. Controls included a PBS injection group. a non-virulent control using *S. epidermidis* ATCC 12228, and a highly-virulent control *S.aureus* ATCC29213. Above, the horizontal lines/points showed the average value of each strain and error bars were SEM.

Table S1. The antimicrobial susceptibility testing result of non-ST42 strains.

ID	Source	MLST	MIC (μg/ml)									
			CIP	GEN	CTX	VAN	ERY	RIF	TET	OXA	CLIN	LIN
**SYSUSHA_71**	Nares	3	128	128	256	1	＞128	＞128	128	＞16	＞32	1
**SYSUGLS069**	Feces	96	256	32	＞256	2	＞128	4	32	＞16	＞32	2
**SYSUSTA_2**	Nares	1	64	16	＞256	1	＞128	＞128	4	＞16	＞32	1
**SYSUSHA_14**	Ward environment	1	4	16	16	1	64	0.125	32	4	8	2
**SYSUSHA_68**	Feces	3	32	16	8	1	128	0.06	4	2	＞32	1
**SYSUSHA_17**	Nares	3	64	256	＞256	1	128	0.125	32	＞16	＞32	2
**SYSUGLS062**	Ward environment	3	64	128	＞256	2	16	＞128	4	＞16	0.125	2
**SYSUSHA_42**	Feces	66	32	128	＞256	1	128	0.06	4	＞16	＞32	1
**SYSUSHA_118**	Nares	104	4	256	＞256	2	＞128	0.125	128	＞16	＞32	2
**SYSUSTA_1**	Ward environment	1	128	64	＞256	1	64	＞128	4	＞16	0.25	1
**SYSUSHA_50**	BAL	1	64	64	＞256	1	＞128	＞128	4	＞16	0.125	1
**SYSUSTA_5**	BAL	3	32	128	＞256	1	64	＞128	4	＞16	0.25	2
**SYSUSTA_4**	BAL	3	32	128	＞256	2	64	＞128	4	＞16	0.25	2
**SYSUSTA_3**	BAL	3	32	128	＞256	2	64	＞128	4	＞16	0.25	2
**SYSUSHA_64**	Nares	3	128	16	8	1	32	0.06	≤1	8	8	1

**SYSUSHA_63**	Feces	3	128	16	8	1	32	0.06	≤1	8	8	1
**SYSUSHA_5**	Nares	3	64	64	＞256	1	64	0.06	4	＞16	＞32	1
**SYSUSHA_9**	Feces	97	32	128	＞256	1	128	0.06	128	＞16	＞32	1
**SYSUSHA_109**	Feces	97	128	128	＞256	2	64	0.125	32	＞16	0.25	2
**SYSUSHA_43**	Feces	112	16	1	8	1	32	0.06	16	4	8	1
**SYSUGLS074**	Ward environment	101	64	256	＞256	2	64	0.125	32	＞16	0.5	2
**SYSUSHA_57**	Nares	1	64	64	＞256	1	16	0.06	≤1	＞16	0.125	1
**SYSUSHA_44**	Nares	1	128	4	8	1	2	＞128	128	1	0.5	1
**SYSUSHA_32**	Feces	1	16	16	4	1	32	0.06	16	0.5	0.125	0.5
**SYSUSHA_58**	Feces	3	16	16	8	1	32	0.06	≤1	4	0.125	1
**SYSUSHA_15**	Nares	3	32	1	16	2	16	0.125	≤1	2	8	2
**SYSUSHA_119**	Feces	3	64	1	16	1	128	0.125	32	8	32	2
**SYSUSHA_33**	Nares	56	8	1	8	2	128	0.125	32	4	＞32	2
**SYSUSHA_101**	Feces	96	128	256	＞256	1	32	0.125	8	＞16	0.125	4
**SYSUGLS045**	Feces	96	128	256	＞256	1	32	0.125	4	＞16	0.25	2
**SYSUSHA_8**	Feces	98	0.25	1	4	0.5	64	0.06	32	1	＞32	1
**SYSUSHA_72**	Throats	99	16	16	4	1	64	0.06	≤1	2	0.25	0.5
**SYSUSHA_70**	Feces	99	16	16	4	1	64	0.06	≤1	2	0.25	0.5
**SYSUSHA_69**	BAL	99	16	16	4	1	128	0.06	≤1	1	0.25	1

**SYSUSHA_29**	Feces	99	64	32	32	1	128	0.125	≤1	4	0.25	2
**SYSUSHA_10**	Ward environment	102	4	1	16	1	64	0.125	32	2	0.125	4
**SYSUSHA_22**	Feces	105	4	1	16	2	64	4	32	1	0.25	2
**SYSUSHA_21**	Nares	107	32	16	16	2	128	0.125	2	4	0.5	2
**SYSUSHA_35**	Nares	109	4	128	4	1	0.5	＞128	≤1	0.25	≤0.06	2
**SYSUGLS064**	Ward environment	-	4	1	8	1	128	0.125	≤1	0.5	8	2
**SYSUSHA_59**	Feces	30	0.25	1	8	0.5	32	0.06	16	4	0.5	0.5
**SYSUSHA_45**	Feces	30	16	8	4	1	0.25	0.06	4	4	0.125	1
**SYSUSHA_41**	Nares	30	0.25	32	2	0.5	32	0.06	16	0.25	0.125	1
**SYSUSHA_34**	Feces	30	16	64	4	0.5	0.25	0.06	≤1	2	0.125	1
**SYSUSHA_27**	Feces	52	4	32	2	1	0.5	0.125	16	0.25	0.25	2
**SYSUSHA_102**	Feces	56	4	1	8	1	128	0.125	2	0.5	0.125	2
**SYSUSHA_30**	Nares	99	16	32	16	2	0.5	0.125	≤1	4	0.125	2
**SYSUSHA_65**	Feces	100	32	1	16	1	32	0.06	≤1	8	0.25	1
**SYSUSHA_108**	Feces	103	16	32	2	2	0.5	0.125	64	0.25	0.25	2
**SYSUSHA_107**	Feces	103	16	64	2	1	0.5	0.125	64	0.25	0.125	2
**SYSUSHA_4**	Feces	105	128	4	4	0.5	32	0.06	≤1	2	0.125	0.5
**SYSUSHA_18**	Feces	105	128	1	16	1	128	0.125	≤1	2	0.25	2
**SYSUSHA_82**	Ward	118	0.25	≤0.125	32	4	64	0.125	64	16	0.125	1

	environment											
**SYSUSHA_28**	Feces	121	128	1	16	0.5	128	0.125	≤1	2	0.125	2
**SYSUSHA_120**	Nares	120	4	1	16	2	64	0.125	≤1	4	0.25	2
**SYSUGLS011**	Ward environment	-	4	128	16	0.5	128	0.125	≤1	1	0.5	2
SYSUSHA_77	Ward environment	117	0.25	1	2	1	64	0.06	≤1	0.25	0.25	1
SYSUSHA_74	Feces	9	0.25	1	8	1	0.5	0.06	128	2	≤0.06	0.5
SYSUSHA_73	Feces	3	16	1	4	1	16	0.06	≤1	2	4	1
SYSUSHA_56	Feces	116	0.5	1	4	1	32	0.06	≤1	0.5	0.125	1
SYSUSHA_55	Feces	1	0.25	8	8	1	128	0.06	≤1	4	0.125	1
SYSUSHA_54	Feces	115	4	1	2	1	16	0.125	≤1	0.25	0.5	2
SYSUSHA_53	Ward environment	122	4	1	8	1	2	2	≤1	1	0.125	2
SYSUSHA_52	Ward environment	114	0.25	1	8	1	0.5	0.06	≤1	4	0.125	1
SYSUSHA_51	Ward environment	-	0.25	1	≤1	0.5	0.5	0.06	≤1	≤0.625	0.125	1
SYSUSHA_47	Ward environment	113	1	1	4	0.5	64	0.06	32	4	0.5	1
SYSUSHA_40	Nares	111	0.5	1	≤1	1	0.25	0.06	2	0.125	0.125	1
SYSUSHA_39	Nares	23	0.5	1	≤1	1	0.25	0.06	≤1	0.125	0.125	1
SYSUSHA_38	Feces	110	4	1	2	1	64	0.125	≤1	0.25	0.5	2

SYSUSHA_37	Feces	52	4	1	2	2	64	0.125	≤1	0.25	0.5	2
SYSUSHA_26	Feces	108	4	1	2	2	0.5	0.125	≤1	0.25	0.25	2
SYSUSHA_25	Feces	52	4	1	2	2	128	0.125	16	0.25	0.25	2
SYSUSHA_24	Nares	30	4	1	16	1	0.5	0.125	≤1	4	0.25	2
SYSUSHA_23	Feces	52	4	1	2	1	64	0.125	≤1	0.25	0.25	2
SYSUSHA_20	Throats	48	4	1	2	1	0.5	0.125	≤1	0.5	0.25	2
SYSUSHA_2	Feces	106	16	1	8	1	0.5	0.125	≤1	2	0.25	2
SYSUSHA_12	Ward environment	-	4	1	16	1	0.5	0.125	≤1	1	0.125	2
SYSUSHA_11	Nares	52	32	1	2	1	0.5	0.125	≤1	0.25	0.5	2
SYSUGLS051	Ward environment	119	4	1	5	1	64	0.126	4	0.25	0.25	2
SYSUGLS031	Feces	30	4	1	16	2	64	0.125	2	2	0.25	2

MLST: Multi-Locus sequence typing, MIC: minimal inhibitory concentratioNares, BAL: bronchoalveolar lavage, ARGs: antibiotic resistant genes.

CIP: ciprofloxacin (Bp 4 ug/mL), OXA: oxacillin (Bp 0.5 ug/mL), CTX: cefotaxime (Bp 4 ug/mL), ERY: erythromycin (Bp 8 ug/mL), RIF: rifampicin (Bp 4 ug/mL), GEN: gentamycin (Bp 16 ug/mL), TET: tetracycline (Bp 16 ug/mL), CLIN: clindamycin (Bp 4 ug/mL), VAN: vancomycin (Bp 32 ug/mL), LIN: linezoid (Bp 8 ug/mL).

Bp: Breakpoint. '-': unidentified ST type. The MDR strain is shown in bold black.

Table S2: The new allelic locus of *S. haemolyticus* ST type.

Id	MLST	arcC	SH_1200	hemH	leuB	SH1431	cfxE	Ribose_ABC
SYSUGLS011	-	14	13	11	19	13?	6	4
SYSUGLS045	96	26	21	1	1	1	1	4
SYSUGLS051	119	26	4	3	33	3	4	23
SYSUGLS064	-	14	13	11	19	13?	6	13
SYSUGLS069	96	26	21	1	1	1	1	4
SYSUGLS074	101	1	1	20	1	1	1	4
SYSUSHA_101	96	26	21	1	1	1	1	4
SYSUSHA_107	103	1	5	1	34	2	1	4
SYSUSHA_108	103	1	5	1	34	2	1	4
SYSUSHA_109	97	19	1	1	1	5	1	4
SYSUSHA_10	102	2	5	1	1	2	17	4
SYSUSHA_118	104	14	6	9	35	9	6	13
SYSUSHA_120	120	6	25	3	4	3	18	22
SYSUSHA_12	-	9	4	8	7	8	4	-
SYSUSHA_18	105	1	5	21	1	2	1	4
SYSUSHA_21	107	1	25	3	4	3	18	22
SYSUSHA_22	105	1	5	21	1	2	1	4
SYSUSHA_26	108	2	1	1	36	1	1	4
SYSUSHA_28	121	13	13	11	18	27	6	8
SYSUSHA_29	99	1	24	22	1	1	1	4
SYSUSHA_2	106	25	24	1	1	1	1	4
SYSUSHA_30	99	1	24	22	1	1	1	4
SYSUSHA_35	109	2	1	1	1	24	1	4
SYSUSHA_38	110	1	5	2	37	5	1	4

SYSUSHA_40	111	1	4	8	7	25	4	4
SYSUSHA_42	66	19	1	1	1	1	1	4
SYSUSHA_43	112	1	1	1	1	26	1	4
SYSUSHA_47	113	14	13	11	19	27	6	8
SYSUSHA_4	105	1	5	21	1	2	1	4
SYSUSHA_51	-	14	13	11	38	13?	6	8
SYSUSHA_52	114	27	1	1	1	2	1	13
SYSUSHA_53	122	14	1	11	3	28	18	4
SYSUSHA_54	115	1	17	13	16	14	6	13
SYSUSHA_56	116	2	5	2	1	2	1	4
SYSUSHA_65	100	13	8	10	17	13	6	3
SYSUSHA_69	99	1	24	22	1	1	1	4
SYSUSHA_70	99	1	24	22	1	1	1	4
SYSUSHA_72	99	1	24	22	1	1	1	4
SYSUSHA_77	117	1	26	9	39	1	1	22
SYSUSHA_82	118	28	4	8	7	8	4	4
SYSUSHA_8	98	1	1	6	1	2	1	4
SYSUSHA_9	97	19	1	1	1	5	1	4

Red indicated the new isoloci identified. MLST: Multi-Locus sequence typing.

Table S4. The biofilm formation result of 97 *S. haemolyticus* strains.

Isolates (n)	Biofilm formation (%)			
	Weak (+)	Moderate (++)	Strong (+++)	Non (0)
Total (97)	29.9 TSB	19.6 TSB	7.2 TSB	43.3 TSB
	42.3 TSB_{Glu}	12.4 TSB_{Glu}	5.6 TSB_{Glu}	40.2 TSB_{Glu}
	17.5 TSB_{NaCl}	8.2 TSB_{NaCl}	2.1 TSB_{NaCl}	68.0 TSB_{NaCl}
ST42 (17)	17.6 TSB	11.8 TSB	5.9 TSB	64.7 TSB
	29.4 TSB_{Glu}	23.5 TSB_{Glu}	5.9 TSB_{Glu}	41.2 TSB_{Glu}
	11.7 TSB_{NaCl}	0 TSB_{NaCl}	17.6 TSB_{NaCl}	70.6 TSB_{NaCl}

Weak (+: ODc < OD ≤ 2x ODc), Moderate (++: 2x ODc < OD ≤ 4x ODc), Strong (+++: 4x ODc< OD), Non (0: OD ≤ ODc).

TSB: only TSB medium; TSB_{Glu}: TSB with 1% glucose; TSB_{NaCl}: TSB with 3% Nacl.
